# Immunomodulatory biomaterials for vascularized and innervated skeletal muscle repair

**DOI:** 10.3389/fimmu.2025.1657015

**Published:** 2025-12-12

**Authors:** Lauren G. Mottel, Brennagh R. Shields, Brian J. Kwee

**Affiliations:** Department of Biomedical Engineering, University of Delaware, Newark, DE, United States

**Keywords:** biomaterials, immunomodulation, muscle regeneration, innervation, vascularization

## Abstract

The repair of functional innervated and vascularized skeletal muscle from severe injuries, such as critical limb ischemia, denervation, and volumetric muscle loss, remains a critical clinical challenge. Regenerative cell therapies are often hindered by donor site morbidities and rapid clearance from injured tissue. Furthermore, emerging tissue engineering and biomaterials approaches are often stifled by–and may even worsen–the chronic, inflammatory microenvironment that debilitates these sites of muscle injury, as well as the underlying peripheral nerves and microvessels. Consequently, the role of the immune system in tissue repair has been increasingly studied and capitalized upon in the design of regenerative biomaterials to overcome these challenges. In this review, recent strategies for the development of immunomodulatory biomaterials for vascularized and innervated skeletal muscle repair will be discussed within the context of muscle, nervous, and vascular tissues, as well as the respective roles of immune cells and tissue progenitors during these repair processes. These strategies span chemical functionalization, sustained presentation of immunomodulatory cues, and inflammatory responses to natural and synthetic biomaterials, among other approaches.

## 1 Introduction

Skeletal muscle is a dynamic tissue that intricately interfaces with the vascular and nervous systems, rendering this tissue responsible for generating voluntary movement and maintaining metabolic homeostasis. A unique hallmark of healthy skeletal muscle is its intrinsic capacity to spontaneously regenerate following minor injury, as muscle regeneration and maintenance are major determinants of quality of life. This regenerative capacity extends beyond muscle fibers to include associated blood vessels and nerves, ensuring full functional recovery. Successful regeneration relies on a regulated inflammatory response, where a coordinated balance between the pro-inflammatory and anti-inflammatory responses is essential. This balance facilitates the clearance of damaged tissue, activation of repair pathways, and resolution of inflammation, ultimately supporting the repair of vascularized, innervated, skeletal muscle. However, in the context of severe injuries, this regenerative process often fails, leading to permanent loss of muscle mass, impaired function, and fibrosis. The persistent pro-inflammatory signals further exacerbate the injury and these subsequent impairments by causing progressive damage to the supplemental vasculature and nerves that are critical to maintaining homeostatic muscle function. Furthermore, these poor outcomes are compounded by factors such as age-related declines in regenerative capacity, insufficient progenitor cell availability, and a dysregulated inflammatory response—all of which limit the muscle’s ability to fully recover and restore function.

Traditional strategies for skeletal muscle repair used in clinical practice include surgical and non-surgical approaches. Surgical approaches are the clinical standard, which include autologous muscle grafts, scar tissue debridement, and minced skeletal tissue transfer (1). While these methods can be effective in restoring structural integrity of the muscle, they often fail to improve muscle function due to donor site morbidity, limited tissue availability, infection, poor revascularization, and insufficient reinnervation (1–3). On the other hand, minimally invasive techniques have become popular, with current clinical practice by often incorporating exercise therapy and administration of anti-inflammatory medications to encourage tissue repair and functional recovery (1, 2). However, these strategies are often hindered by the short half-lives, systemic effects, and non-specificity of anti-inflammatory drugs in their efforts to dampen the immune system (4, 5). Meanwhile, cell based therapies–particularly those involving muscle stem cell transplantation–remain under investigation in clinical trials and have not yet been widely adopted in standard care due to challenges with poor cell survival, functional integration, and limited long-term efficacy of transplanted cells within the injured tissue microenvironment (5, 6).

Emerging tissue-engineering approaches combining growth factors, cells, scaffolds, and/or drug delivery materials have shown promising preclinical efficacy in promoting skeletal muscle repair (7, 8). Localized delivery of soluble cues from drug delivery materials can promote muscle regeneration by modulating the cellular microenvironments and by stimulating endogenous tissue repair. Tissue engineered constructs, including scaffolds loaded with growth factors or cells, enable *de novo* formation of skeletal muscle tissue and their corresponding vasculature and nerves *in vitro* and *in vivo.* These therapies can be utilized to treat volumetric muscle loss (VML)—a severe injury marked by the traumatic and/or surgical loss of a critical volume of skeletal muscle that causes permanent functional impairment and long-term disability (9). Similar strategies have also been utilized to promote regeneration of blood vessels and nerves as individual tissue types, but ongoing work is needed to attain the multi-tissue repair necessary for vascularized and innervated skeletal muscle. Currently, these approaches often provoke unwanted inflammatory responses that prevent integration with the host tissue, inhibit regeneration, and fail to restore pre-VML functionality (3, 8). Long-term translational challenges include fibrotic scarring and scalability of biomaterial or tissue design (2, 5). Thus, there is an unmet need for therapeutic strategies with increased efficacy to achieve functional, vascularized and innervated skeletal muscle repair.

A common challenge among both clinical and preclinical muscle, nerve, and blood vessel regeneration approaches is the chronic inflammatory microenvironment that is present at sites of severe muscle injuries and diseases, which can prevent engineered materials and therapies from achieving their regenerative potential. Severe muscle injuries and diseases—such as critical limb ischemia, muscular dystrophies, denervation, and volumetric muscle loss—are often marked by rampant inflammation that impairs the spontaneous regeneration of damaged muscle, vascular, and nervous tissues. If left unchecked, such inflammation can rapidly devolve from symptoms of muscle and/or nerve pain into significant loss of mobility and functional decline. Prolonged pro-inflammatory responses further contribute to fibrosis, fatty degeneration, and impaired regeneration of the muscle (10, 11). Moreover, impaired adaptive immunity due to age perpetuates this degenerative inflammation and diminishes regeneration (12). Therefore, there is a critical need for immunomodulatory therapeutic strategies to repair these impacted tissues and restore their integrated functions.

Consequently, immune cells have presented themselves as prospective cellular targets to coordinate repair and improve therapeutic efficacy. As both innate and adaptive immune cells—such as neutrophils, macrophages, and T cells—work in concert with progenitor cells during tissue healing, their respective functions may be modulated to drive different stages of the repair process. As such, engineering approaches have begun to adapt the physical and chemical cues of biomaterial designs to overcome the limitations of traditional tissue repair strategies and to leverage a restorative immune response. In this review, we will discuss recent advances in the development of immunomodulatory biomaterials for vascularized and innervated skeletal muscle repair, placed within the context of tissue-specific regeneration and the roles immune cells play in these respective processes. Specifically, we will review different biomaterial approaches that target innate and/or adaptive immune cells, such as material-based delivery of immunomodulatory cues and cells, material-functionalization, modulation of biomaterial mechanical properties, and utilization of intrinsic inflammatory responses to synthetic and naturally-derived materials. We will highlight biomaterial approaches that not only mediate direct muscle repair, but also those that promote the necessary vascularization and innervation to sustain regenerating muscle tissue. The scope of our discussion will focus primarily on key findings from pre-clinical animal studies, which provide insight into the potential of immunomodulatory biomaterials for functional skeletal muscle, nerve, and vascular repair.

## 2 Physiological overview of skeletal muscle regeneration

Skeletal muscle is the most abundant tissue in the human body by mass, responsible for regulating posture, respiration, and voluntary movements among other functions (13). As such, skeletal muscle is a highly organized and intricate tissue to support these diverse functions. Skeletal muscle is primarily composed of multinucleated, striated bundles of myofibers (14). Each myofiber is encased by a layer of connective tissue, the endomysium, and the sarcolemma, a membrane that possesses complex proteins which aid in contraction. Among these proteins include dystrophin, whose absence in disorders such as Duchenne’s muscular dystrophy result in muscle weakness and atrophy (15). Each myofiber is also supported by a system of capillaries and nerves found throughout the endomysium, enabling exchange of nutrients for muscle fiber homeostasis as well as innervation to support voluntary muscle contraction. Similarly, bundles of myofibers known as fascicles are encased in their own connective tissue, before compartments of fascicles organize into muscle tissue. Skeletal muscle also contains resident adult stem cells known as muscle satellite cells that are critical for growth, repair, and regeneration. These satellite cells interface with not only surrounding muscle fibers but with infiltrating immune cells and other interstitial resident cells (14). Therefore, to rationally design immunomodulatory biomaterials to promote muscle regeneration, it is first important to highlight the relevant progenitor and immune cell populations—and their respective dynamics—that are critical for tissue homeostasis and repair, as they may serve as potent cellular targets.

### 2.1 Key progenitor cells

#### 2.1.1 Satellite cells

Residing beneath the basement membrane of each myofiber exists a quiescent population of muscle stem cells, or muscle satellite cells (MuSCs), that are responsible for the spontaneous regeneration of muscle. In response to injury, MuSCs either become activated myoblasts or self-renew to maintain the quiescent MuSC reservoir to prepare for potential future muscle damage (16). After differentiating into myoblasts, the myoblasts proliferate and convert into myocytes that subsequently fuse into myotubes to form new myofibers that integrate with surviving myofibers and promote repair. These dynamic processes of self-renewal and differentiation are highly regulated by 1) key transcriptional factors such as paired box 7 (Pax7)—which is uniformly expressed by all MuSCs as an indicator of their stemness, 2) growth factors like amphiregulin (AREG) that directly stimulate MuSCs, and 3) myogenic regulatory factors such as myoblast determination protein 1 (MyoD1) that control the differentiation of activated MuSCs (17, 18). However, in the case of chronic or severe injuries marked by degenerative inflammation, these processes become disrupted: MuSCs are susceptible to exhaustion and reduced potency as the dormant MuSC pool becomes repeatedly activated to repair continuous injury and are forced to forego self-renewal (19). This sensitivity to intracellular damage and exhaustion also persists with age as damage accumulates over a lifetime, which further exacerbates loss of MuSC number and function. This highlights a critical need to address chronic muscle injuries in our growing aged population (17).

#### 2.1.2 Fibroadipogenic progenitors

In addition to MuSCs, fibroadipogenic progenitors (FAPs) have demonstrated a significant role in muscle regeneration. FAPs can be categorized as mesenchymal progenitors, with the capacity to differentiate into either fibroblasts or adipocytes. FAPs typically reside undifferentiated in the stromal space between resting myofibers (16). Concomitant with MuSC activation and proliferation, FAPs also expand following skeletal muscle injury. In fact, FAPs regulate MuSC expansion and myogenic differentiation via expression of factors such as interleukin(IL)-6 and insulin-like growth factor 1 (IGF-1). Further, FAPs also contribute to homeostatic maintenance, as depletion of FAPs in a murine model led to muscle atrophy and MuSC reduction (16), (20). Conversely, however, FAP’s bipotential also renders the cells responsible for detrimental fatty and fibrotic infiltration in degenerating muscles, both in chronic conditions such as muscular dystrophy and during the normal aging process. Recent studies have demonstrated that FAPs engage in inflammatory crosstalk during muscle repair, implicating immune cells as potential cellular targets to modulate FAP function. The secretion of the chemokine IL-33 by FAPs established a pro-regenerative niche, increasing regulatory T cell (Treg) accumulation and promoting repair of aged muscle (21), while activation of type 2 IL-4/IL-13 signaling promoted FAP proliferation and downstream clearance of necrotic debris while inhibiting their differentiation into adipocytes (16). Thus, regulation of FAP dynamics in tandem with that of MuSCs is critical to ensure functional muscle regeneration.

### 2.2 Notable immune cells

For muscle to spontaneously regenerate, a balanced inflammatory response of pro-inflammatory and anti-inflammatory immune cells must work in concert with MuSCs. Pro-inflammatory immune cells, such as neutrophils, first clear tissue debris and induce the proliferation of MuSCs before anti-inflammatory cells, such as M2 macrophages, enhance remodeling and maturation of developing muscle fibers (10). Several immune cells in the inflammatory response secrete cytokines that may regulate MuSC function and downstream skeletal muscle repair. The most prominent cellular targets for the development of immunomodulatory biomaterials will be discussed below.

#### 2.2.1 Neutrophils

As the primary responders to injury, neutrophils are responsible for host defense against pathogens and for phagocytosis of necrotic muscle tissue. In tandem with M1 macrophages, neutrophils secrete proinflammatory cytokines that encourage MuSC proliferation (9). Notably, when primary muscle progenitor cells were cultured with conditioned media from activated neutrophils, there was a significant increase in the number of proliferating muscle progenitor cells due to neutrophil-secreted factors such as CCL3/CCL4 and MMP-9; however, the prolonged presence of these factors significantly reduced differentiation, implicating impaired myogenesis (22). The capacity of neutrophils to enhance early stages and impede later stages of myogenesis is reflective of how an orchestrated and balanced inflammatory response is necessary for recovery from severe injury. The findings of these *in vitro* studies have since been corroborated *in vivo*. In a comparative study of volumetric muscle loss injuries resulting in regeneration or fibrosis, it was found that an imbalance between neutrophils and natural killer cells impaired MuSC-mediated regeneration. Specifically, prolonged presence of neutrophils decreased MuSC fusion and increased TGF-β signaling in the muscle defect, the latter of which is known to inhibit natural killer cell cytotoxicity (9).

#### 2.2.2 Macrophages

Macrophages possess a dynamic role in the progression of skeletal muscle repair and tissue homeostasis due in part to the broad spectrum of their phenotypic functions. Initially, circulating monocytes are alerted to injury sites by invading neutrophils. Upon extravasation into a damaged microenvironment enriched with proinflammatory cytokines such as IFN-γ and tumor necrosis factor, the monocytes become activated and differentiate into macrophages. The initial pro-inflammatory M1 macrophages serve to clear necrotic debris and stimulate myoblast proliferation; afterwards, these cells transition to an M2 phenotype associated with elevated IL-10 levels to promote MuSC differentiation and muscle fiber maturation—a critical junction for normal regeneration (23). Within the context of severe ischemic injuries, analysis of murine and human ischemic muscles revealed that injured tissues were enriched with pro-inflammatory macrophage signatures and premature differentiation of MuSCs that could not coordinate repair (24). Furthermore, macrophage depletion at the time of peak macrophage phenotype switching in regenerating muscle significantly impaired muscle growth and disrupted regenerative transcription factor expression (25, 26). However, a recent study analyzing the effects of muscle defect size (regenerative 2 mm defects versus fibrotic 4 mm defects) demonstrated that macrophages found in regenerative outcomes and macrophages in fibrotic outcomes exhibited similar temporal expression profiles of M1 and M2 macrophage phenotypes but at different magnitudes of expression. The fibrotic defect macrophages did not appear “more pro-inflammatory” than the regenerative macrophages by the typical M1/M2 macrophage markers. Rather, the fibrotic defect macrophages exhibited defective regenerative function by gene expression (27). Thus, the spectrum of regenerative and fibrotic macrophages is likely more complex than the traditional M1/M2 macrophage paradigm.

#### 2.2.3 T cells

T cells, consisting of CD4^+^ helper and CD8^+^ cytotoxic phenotypes, have also been shown to mediate repair by secreting a wide variety of factors—such as IFN-γ, TGF-β, and IL-4, which exhibits control over macrophages and MuSCs alike amidst muscle repair. In a study seeking to replicate the endogenous microenvironment for long-term MuSC culture and expansion, Fu et al. demonstrated that T cell-derived cytokines (particularly a combination of IL-1α, IL-13, TNF-α, and IFN-γ) were critical for stimulating MuSC proliferation *in vivo* following injury and promoted serial expansion of MuSCs *in vitro* beyond 20 passages (28). However, an overabundance of T cells at injury sites can prove harmful, as demonstrated by Kohno et al. In a cardiotoxin muscle injury model in ubiquitin ligase deficient mice, CD8^+^ T cell presence was left unchecked and persisted for two weeks post-injury, resulting in increased fibrotic deposition and dysfunctional repair (29). This is mimetic of the deleterious inflammation found in severe muscle injuries like critical limb ischemia.

Furthermore, emerging evidence implicates that infiltrating regulatory T cells (Tregs) are critical for tissue remodeling following ischemic injury by secreting factors which directly stimulate muscle repair (30). In models of muscular dystrophy or acute muscle injury, the ablation of Tregs exacerbates injury and inflammation, resulting in impaired regeneration (31, 32). These studies have revealed a subpopulation of tissue-specific Tregs, distinct from conventional lymphoid-derived Tregs, which additionally secrete factors that directly stimulate tissue repair. Particularly, muscle-specific Tregs accumulate at sites of muscle injury and secrete the growth factor amphiregulin to induce MuSC differentiation into muscle fibers (31) and to promote Treg-mediated modulation of inflammation and fibrosis.

## 3 Design of immunomodulatory biomaterials for skeletal muscle regeneration

### 3.1 Biomaterial identity regulates innate immune responses

One facet of biomaterials that shapes their immunomodulatory potential at muscle injury sites is the chemical composition of the base biomaterial itself. Particularly, the differential use of synthetic and naturally derived materials for scaffolds have been investigated to examine their respective inflammatory responses and downstream regulation of recruited immune cells. Delineating the impact of different biomaterials on innate immune cells is a common route in immunomodulatory design, due to their first-line response to injury and orchestration of inflammation. Notably, Sadtler et al. demonstrated divergent myeloid responses between tissue-derived, particulate extracellular matrix (ECM) and synthetic particulate polyethylene (PE) and polyethylene glycol (PEG) hydrogel particles as scaffolds in a muscle wound microenvironment (33). Initial neutrophil infiltration was largely modulated by material stiffness and particle size of synthetic scaffolds. Larger diameter particles recruited fewer macrophages and were less encapsulated in CD11b^+^ Ly6G^+^ neutrophil coronas when interfacing with muscle tissue compared to smaller particles, while stiffer PEG hydrogels induce chronic recruitment of granulocytes. Furthermore, synthetic PE scaffolds induced chronic neutrophilic infiltrate at the injury site, leading to increased fibrosis. The response to the synthetic materials greatly diverged from the response to biologic ECM materials. ECM scaffolds predominantly recruited Th2 adaptive immune cells and upregulated CD206 expression on macrophages in hand with new muscle fiber formation, while synthetic scaffolds induced prolonged and dense neutrophil infiltrate and loss of Type 2 markers on macrophages. The authors do note that the physiochemical state of these biomaterials may impact the divergent response observed in their study, as compared with other studies comparing natural biomaterials to synthetic biomaterials. The ECM particles in this study were uncross-linked, whereas cross-linked ECM scaffolds may induce more fibrotic capsulate formation. Synthetic materials, such as PEG, induce varying foreign body responses based on cross-linking density, stiffness, and surface modification. Regardless, the design and eventual clinical translation of natural or synthetic materials for skeletal muscle regeneration must remain contingent on dampening undesirable and overactive inflammation, not just broadly promoting higher immune cell responses.

These fundamentally divergent immune responses to natural and synthetic materials based on their composition have also been leveraged to identify distinct immune populations. Sommerfeld et al. utilized a murine bilateral traumatic muscle defect model and conducted extensive single-cell RNA sequencing to characterize functional macrophage subpopulations in biologic matrices (urinary bladder ECM) and synthetic polycaprolactone (PCL) biomaterials to represent pro-regenerative and pro-fibrotic microenvironments, respectively (34). Specifically, a new population of macrophages were identified at fibrotic deposition sites possessing Type 17 and autoimmune signatures. ECM scaffolds demonstrated greater macrophage heterogeneity associated with tissue repair phenotypes, whereas PCL-treated wounds demonstrated reduced macrophage heterogeneity associated with interferon cytokine production and autoimmune receptors.

**Figure 1:**
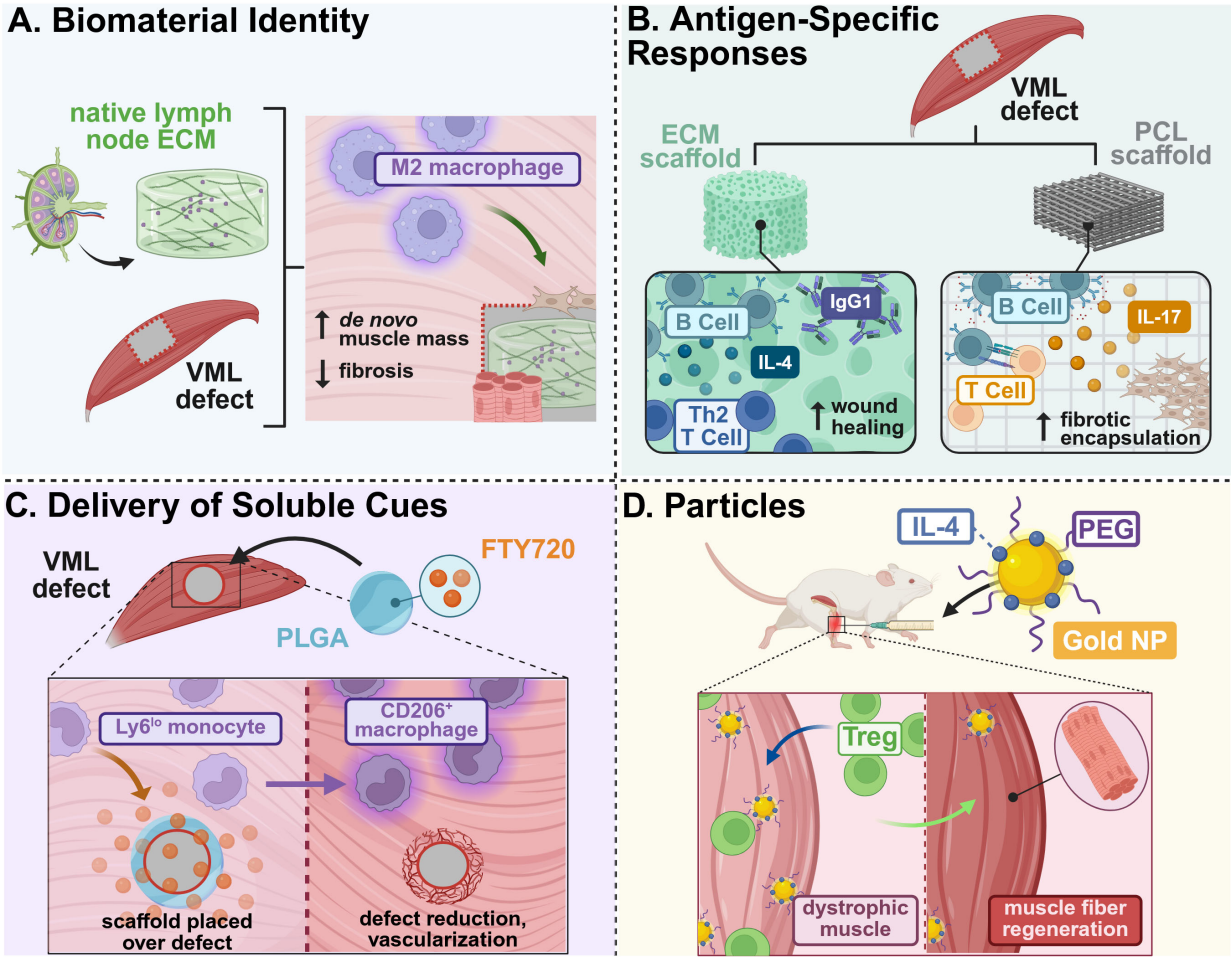
Example strategies of immunomodulatory biomaterial designed for skeletal muscle tissue regeneration. **(A)** Lymph node ECM-derived hydrogels promoted M2 macrophage polarization and recruitment to a VML defect, enhancing *de novo* muscle growth and reducing fibrotic deposition (35). **(B)** The presence of antigens on biologic ECM scaffolds promoted early B cell recruitment and maturation, as compared to PCL scaffolds that increased B cell antigen presentation and foreign body responses, as well as promoted Th2 T cell-driven tissue remodeling and functional regeneration (36, 37). **(C)** Delivery of soluble FTY720, an immunosuppressive lipid mediator, from PLGA films increased recruitment of anti-inflammatory monocytes that resulted in defect reduction and vascularization in VML injuries (38). **(D)** Intramuscular injection of IL-4-conjugated gold nanoparticles enhanced regulatory T cell recruitment and muscle fiber regeneration in dystrophic-mimetic muscle in aged mice (39). This figure was created in BioRender. Mottel, L. (2025) https://BioRender.com/nmrb11e .

Incorporating ECM from immune organs such as lymph nodes is also attractive for directing immunity by mimicking lymphatic microenvironments. To mitigate detrimental inflammatory host defense mechanisms in response to medical implants, Choi et al. developed a lymph node ECM-derived (LNEM) platform modulating macrophage phenotype in both 2D and 3D systems. Notably, implanted 3D LNEM hydrogel significantly enhanced M2 macrophage phenotype and muscle function recovery in a murine VML model with increased *de novo* muscle mass as compared to 3D collagen hydrogel controls (35) (**Figure 1A**).

Furthermore, skeletal muscle-derived ECM itself is a potent immunomodulatory therapeutic for severe muscle injuries due to its close alignment with the microenvironment of the injury site. For example, an acellular, injectable skeletal muscle (SKM) ECM hydrogel was developed to preserve muscle content and reduce tongue fibrosis following partial glossectomy towards treatment of dysphagia. This SKM hydrogel improved muscle regeneration within the scarred region, as quantified by immunohistochemical staining and a shift from pro- to anti-inflammatory macrophage phenotypes assessed via gene expression signatures. Specifically, SKM-induced tongue repair was associated with FAP-directed immunomodulation due to increased *Il33* and *Il6* gene expression—a primary means of regulating muscle stem cell function, as well as neovascularization of the tongue muscle tissue (40).

### 3.2 Antigen-specific responses to ECM materials

Additional physio-chemical cues beyond the selection of the base material have been investigated in pursuit of promoting immuno-directed skeletal muscle repair. Specifically, antigen-specific responses to the ECM-derived materials from various tissue sources have been explored across several innate and adaptive immune cell populations. Through application of a pro-regenerative decellularized ECM in a VML model, Lokwani et al. identified a distinct population of trauma-associated dendritic cells (tDCs) accumulating in ECM treatment groups that promoted immunoregulatory crosstalk with T-cell activation and macrophage polarization. There was also significant upregulation of both XCL-1 (a NK-produced chemokine for the XCR1 receptor on conventional DCs) and E-cadherin (which is associated with M2 macrophage remodeling) in muscle sites for ECM-treated groups, implicating potential mechanisms of DC recruitment. In the absence of tDCs, there was increased inflammation and progressive calcification of muscle tissue and vice versa with tDCs recruited from ECM biomaterials; this demonstrated tDCs as key antigen-presenting cells for muscle tissue recovery post-injury (41).

Beyond the innate immune response, there has been increasing investigation into the long-term regenerative impact of biomaterial composition on adaptive immunity. For example, Moore et al. compared B cell responses to decellularized, small intestinal submucosal ECM and particulate polycaprolactone (PCL) in a murine model of volumetric muscle loss (VML) to probe B cells’ regenerative function. Their respective material composition greatly influenced B cell phenotype in both regional lymph nodes and the spleen, wherein ECM scaffolds promoted earlier B cell recruitment within the wound and germinal center formation in draining lymph nodes; in contrast, PCL materials demonstrated prolonged B cell presence and antigen presentation in the injury site, which contributed to increased fibrotic deposition (36) (**Figure 1B**). This study highlighted the interplay between local implant composition and systemic immunological factors, which may need to be considered in the advancement of scaffolding strategies, particularly in targeting adaptive immune populations. The use of both bone and cardiac muscle-derived ECM scaffolds were also utilized in a VML model to evaluate their respective regenerative potential. In Rag1^-/-^ mice (deficient in mature T and B cells), loss of IL-4 regulation suggested a Th2 driven scaffold immune environment. These Th2 T cells were associated with M2 macrophage phenotype during tissue remodeling and promoted functional tissue regeneration in both treadmill and histological analyses (37) (**Figure 1B**). Designing biomaterials which leverage this Th2-specific response to ECM scaffolds may also support the advancement of therapies that target adaptive immunity and subsequent systemic and local regeneration.

### 3.3 Delivery of soluble cues

In addition to leveraging cellular responses to a base biomaterial to induce downstream immunomodulatory effects, the delivery of soluble cues to directly stimulate immune cells has also been explored to support skeletal muscle regeneration. Han et al. identified that senescent T-cell signaling promotes high levels of IL-17-associated Type 3 immunity and adipogenesis, which significantly impairs the pro-regenerative capacity of ECM materials in a VML model in aged mice. This has great implications in developing treatments for our growing elderly population, who are most susceptible to severe muscle injuries. To overcome these setbacks, a combination therapy of ECM materials with soluble IL-17-neutralizing antibodies was implemented, resulting in an increased prevalence of regenerating muscle fibers (42). Other soluble cues, besides antibodies and drugs, have been explored in pursuit of directing skeletal muscle repair. A regenerative immunotherapy derived from helminth soluble egg antigens (rSEA) was developed by Maestas et al. to stimulate production of IL-4 and type-2 cytokines without infection cascades (43). Helminth infections, surprisingly, have reduced symptom severity in autoimmune diseases due to potential alterations in the microbiome and immunomodulatory behavior, and are further associated with increased IL-4Rs. The authors evaluated this helminth-derived immunotherapy in a VML model, wherein bolus administration of rSEA increased IL-4-expressing eosinophils, Th2 T cells, and Tregs, correlating with increased muscle repair. Furthermore, Bartolacci et al. sought to leverage the immunomodulatory effects of IL-33-laden matrix-bound nanovesicles (MBVs) from the ECM by administering exogenous MBVs in the muscles of cardiotoxin-injured *il33*^-/-^ mice. MBV-delivered IL-33 stimulated differentiation of MuSCs *in vitro* and promoted pro-reparative CD206^+^ macrophages and partial functional muscle recovery following injury *in vivo*, independent of the traditional IL-33:ST2 axis affiliated with Treg recruitment and muscle repair (44).

The sustained presentation of soluble cues and cytokines from biomaterials that locally modulate distinct immune cell populations may prove advantageous in further boosting therapeutic efficacy in coordinating skeletal muscle repair. The local delivery of FTY720 (an agonist of sphingosine-1-phosphate GPCR 3 (S1PR3) signaling lipid produced by RBCs) from poly lactic-co-glycolic acid (PLGA)-based biomaterials increased anti-inflammatory monocytes in a novel spinotrapezius VML model, which increased the overall kinetics of wound healing and growth of muscle fibers (38) (**Figure 1C**). In addition to factors that traditionally polarize macrophages, an injectable, heparin-based microparticle platform was designed to locally deliver stromal cell-derived factor-1alpha (SDF-1α) following rotator cuff injury in rats; SDF-1α was previously associated with CXCR4 chemotactic gradients and can potentially recruit pro-regenerative cell populations. One week following administration, there was a 4.3-fold increase in M2-like macrophages and 3-fold increase in mesenchymal stem cells in microparticle groups versus injury alone, suggesting a shift to a more pro-regenerative microenvironment at the injury site (45).

Particle-based approaches have also been leveraged as vehicles for the delivery of different immunomodulatory, soluble cues for skeletal muscle repair. IL-4-conjugated gold nanoparticles were administered following hindlimb ischemia injuries to induce tissue repair via macrophage phenotype switching. These IL-4 NPs locally induced M2 macrophage polarization and induced a 40% increase in muscle force in injured muscle compared to NPs alone, while soluble IL-4 proved ineffective in directing macrophage polarization (46). Expanding on the success of this work, either IL-4 or IL-10 were each conjugated onto gold nanoparticles (PA4, PA10) and injected into chronically injured, aged, dystrophic mice to evaluate whether anti-inflammatory modulation to repair muscle tissue occurred. PA4 enhanced muscle fiber area and contractile force of injured muscle due to increased Treg cell infiltration (39) (**Figure 1D**).

## 4 Physiological overview of nerve regeneration

Proper innervation is essential for effective skeletal muscle repair. Without innervation, muscle tissue cannot generate contractile forces required for coordinated movement and functional recovery. Peripheral nerves are dynamic structures responsible for transmitting electrical signals that facilitate both sensory perception and motor control. Peripheral nerves are composed of neurons, glial cells, and microvessels, which provide nutrients to the tissue and help maintain homeostasis. Schwann cells are the primary glial cells of the peripheral nervous system. Schwann cells are crucial to the function of peripheral nerves, forming the myelin sheath. The myelin sheath insulates the axon by providing protection and by enabling signal transmission to the neuromuscular junction (NMJ), where the motor neurons interface with muscle fibers. This synaptic connection allows for skeletal muscle to respond to nerve impulses, initiating muscle movement. Disruption of this neural pathway impairs both the structural and functional integrity of the muscle tissue, resulting in muscle atrophy, muscle degeneration, and loss of sensorimotor function. During regeneration in severe muscle injuries, newly formed muscle fibers also require reinnervation and formation of NMJs to restore functionality. While muscle and nerve are distinct tissue types, they are functionally dependent on each other, as damage to one tissue drives degeneration in the other.

### 4.1 Key progenitor cells

#### 4.1.1 Satellite glial cells

Satellite Glial Cells (SGCs) wrap tightly around the cell body of neurons, forming a protective sheath at the interface of the central and peripheral nervous system, helping to transmit signals from the dorsal root ganglia (DRG) to the periphery. Although historically less studied than other progenitor cells, SGCs are becoming increasingly recognized for their regenerative potential. *In vitro* experiments have shown that SGCs express CDH19, a marker uniquely associated with SGC precursors. SGCs also express GAP43, a plasticity protein, and BFABP, a protein required for neuronal migration, which are both expressed in high levels by SGCs during the developmental stage. The expression of SGC associated markers is crucial to nerve repair as the markers are involved in the regulation of myelin synthesis and maintenance, ensuring proper nerve function (47). SGCs express glial fibrillary acidic protein (GFAP), which is associated with the modulation of inflammation after injury (48). Notably, SGCs contribute to regeneration through the activation of the Peroxisome Proliferator Activated Receptor-alpha (PPAR) pathway in sensory neurons, a process involved in regulating inflammation and neuronal recovery within the functional nerve unit (49, 50).

#### 4.1.2 Schwann cells

In addition to forming the myelin sheath, Schwann Cells (SCs) orchestrate repair after peripheral nerve injury (PNI). SCs exist in both non-myelinating and myelinating phenotypes, and these cells possess incredible plasticity. Upon injury, SCs undergo phenotypic changes mediated by the transcription factor c-Jun, which is essential for Wallerian Degeneration, the process by which injured axons and myelin are broken down distal to the site of injury; this mechanism is essential for the clearance of debris, the activation of the repair process, and the emergence of the repair Schwann Cell. Unlike normal SCs that maintain myelin and support axons, repair SCs promote regeneration by clearing damaged myelin, recruiting macrophages, supporting the survival of damaged neurons, secreting factors that stimulate axon growth, and guiding axons back to their targets (51, 52). During repair, SCs proliferate, guide axonal growth, and secrete a variety of regenerative biological factors, including nerve growth factor (NGF), brain-derived neurotrophic factor (BDNF), amphiregulin (AREG), and interleukin-10 (IL-10) (53).

### 4.2 Notable immune cells

Immune cells play a pivotal role in nerve regeneration by regulating the inflammatory response and creating a microenvironment conducive for tissue repair. Both the innate and adaptive immune systems contribute to this complex process. In particular, macrophages and T-cells, including Tregs, are key players that secrete bioactive factors that influence progenitor cell activity, axonal regeneration, and remyelination.

#### 4.2.1 T-cells

CD4^+^ helper T cells are involved in initiating Wallerian Degeneration. Th1 T cells secrete interferon-gamma (IFN-γ), a proinflammatory cytokine that is necessary for immune cell activation but can also inhibit nerve regeneration (54). In contrast, Th2 T cells secrete anti-inflammatory cytokines, such as IL-10, IL-4, and IL-13, that promote axon regeneration and remyelination (54).

Tregs promote nerve repair by suppressing excessive inflammation at the site of injury. Following peripheral nerve injury, Tregs infiltrate the injury site and secrete anti-inflammatory cytokines, such as IL-10, to suppress proinflammatory Th1 responses by inhibiting the production of IFN-γ (54, 55). This immunomodulatory function of Tregs reduces neuronal damage and prevents the development of neuropathic pain (54). *In vivo* studies have shown that the local delivery of Tregs enhances regeneration by dampening immune activation, thus promoting tissue remodeling in complex nerve architectures (56). Tregs also contribute to regeneration by secretion of cellular communication network factor 3 (CCN3), a matricellular protein that enhances oligodendrocyte differentiation and promotes remyelination specifically in the central nervous system (57). Furthermore, Tregs influence the phenotype of innate immune cells. For example, Tregs influence M2 macrophage polarization in the DRG, thus promoting an anti-inflammatory microenvironment and minimizing the development of pain after nerve injury (58).

#### 4.2.2 Macrophages

Macrophages are the main drivers of Wallerian Degeneration. Macrophages are recruited to the site of nerve injury, where they phagocytose cellular debris and release cytokines, such as IL-6, IL-1α, TNF-α, IL-10, and IL-4, that transform the regenerative microenvironment (59, 60). In later stages of regeneration, macrophages regulate SC differentiation by secreting factors that support the transition to the myelinating phenotype. When macrophages are depleted, remyelination is impaired and immature SCs persist, highlighting macrophages’ essential role in driving functional recovery after injury (61). Additionally, macrophages promote angiogenesis by secreting vascular endothelial growth factor (VEGF). VEGF promotes the growth of new blood vessels, which enhances SC infiltration in the injury site. The newly formed microvasculature not only supports tissue viability but also serves as tracks to guide SC migration across nerve gaps, facilitating axonal regrowth (62).

#### 4.2.3 Key growth and immunomodulatory factors

Multiple molecular factors coordinate the complex process of peripheral nerve repair by modulating immune responses and supporting cellular regeneration. Nerve growth factor (NGF) plays a key role in the early stages of repair by promoting neuron survival, stimulating Schwann cell differentiation, and enhancing axon and myelin regeneration (63, 64). Vascular endothelial growth factor (VEGF) supports neovascularization and Schwann cell invasion, while also boosting phagocytic activity of microglia and SCs in inflammatory environments to facilitate tissue remodeling (65, 66). Amphiregulin (AREG), an anti-inflammatory cytokine upregulated after injury, promotes Schwann cell proliferation and accelerates neurite outgrowth and axon elongation (67). Interleukin-10 (IL-10) enhances nerve regeneration by promoting anti-inflammatory macrophage polarization and increasing Schwann cell activation, thus contributing to reduced inflammation and improved sensory-motor recovery following injury (68–70).

## 5 Design of immunomodulatory biomaterials for peripheral nerve regeneration

### 5.1 Chemical cues guiding peripheral nerve repair

Chemical cues in the form of selective polymer and metal incorporation into biomaterial scaffolds can significantly influence the immune microenvironment following peripheral nerve injury. Infiltrating immune cell populations interact with the chemical cues in these biomaterials, which induces cellular changes and promotes immune cell activity. Chemical cues not only affect immune cell behavior, but can also modulate interactions with key progenitor cells, such as SCs, enhancing processes such as myelination, axonal guidance, and tissue integration essential for nerve repair.

Chitosan, a naturally derived polysaccharide known for its biocompatibility, biodegradability, and immunomodulatory properties, is commonly used as a scaffold in nerve regeneration. One application of chitosan includes the development of chitosan nerve grafts (CNGs), which were assessed in a delayed reconstruction model involving critical length sciatic nerve injuries in rats. In this study, two-chambered chitosan film enhanced chitosan nerve guides (CFeCNGs)—in which a perforated chitosan film was introduced longitudinally into the hollow nerve guidance structure—were evaluated for their regenerative effect. The architecture of the CFeCNG had no effect on regeneration in the delayed repair setting, but the presence of chitosan within the nerve guidance channel facilitated immune modulation. Specifically, the addition of the chitosan film promoted the activation and polarization of monocytes into M2 macrophage phenotypes compared to the hollow chitosan nerve guidance channel (hCNG). This phenotypic switch promoted a pro-regenerative tissue microenvironment that supported tissue remodeling and Schwann Cell recruitment and activity (71). To evaluate the effect of CFeCNGs on muscle reinnervation, the compound muscle action potential (CMAP) was recorded in the tibialis anterior and plantar interosseus muscles. At 150 days post-injury, the tibialis anterior muscle demonstrated increased reinnervation of motor endplates in the CFeCNG group (71).

Similarly, aligned nanofibrous nerve guidance conduits made of poly(lactic-co-glycolic acid) (PLGA) with chitosan was developed with electrospinning, which was further enhanced with a polydopamine (PDA) coating. The resulting scaffold demonstrated dual functionality; the aligned nanofiber architecture provided guidance for the direction of SC migration, while the PDA coating increased cellular adhesion and influenced the immune microenvironment through multiple mechanisms. The material locally modulated the expression of bioactive molecules such as fibroblast growth factor (FGF), while also modulating reactive oxygen species (ROS) levels. The modulation of ROS levels by the material induced a switch to the pro-healing M2 macrophage phenotype and promoted an immune microenvironment conducive for repair in long-segment peripheral nerve injuries in rats. After 12 weeks, motor function as a mode of reinnervation was evaluated by the sciatic functional index, electrophysiological testing, muscle fiber staining, and muscle weight analysis. The PLGA/CS-PDA-bFGF group exhibited higher sciatic functional index scores which corresponded with higher compound muscle action potential amplitudes. Additionally, muscle fibers in the PLGA/CS-PA-bGFG groups had greater muscle mass and exhibited more uniform fiber morphology, indicating that treatment promoted nerve regeneration that effectively supported muscle repair (72).

**Figure 2:**
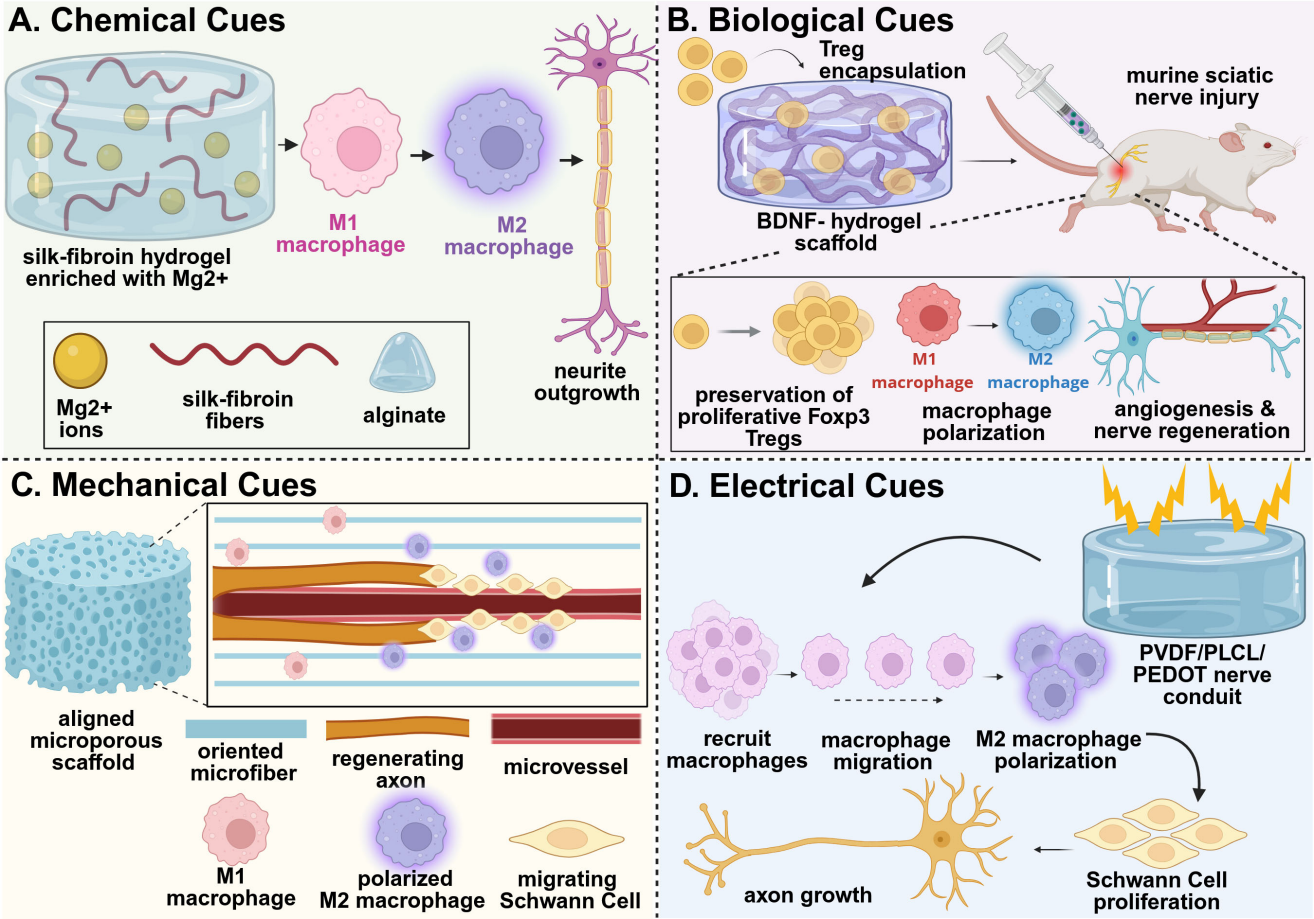
Example strategies of immunomodulatory biomaterial design for nerve tissue regeneration. **(A)** An adaptable hydrogel network composed of silk-fibroin/alginate network releasing magnesium ions promoted macrophage polarization to their pro-healing M2 phenotype, supporting a regenerative microenvironment that effectively prevented muscle atrophy and promoted nerve regeneration *in vivo* (73). **(B)** A self-assembled peptide chitosan hydrogel encapsulating Tregs preserved the proliferative phenotype of the Tregs, thus promoting macrophage polarization and directly stimulating nerve vascularization, regeneration, myelination, and functional recovery *in vivo* (74). **(C)** Aligned P(MMd-co-LA) nanofibers regulated the polarization of macrophages to their M2 phenotype and reduced expression of inflammatory factors within the injured nerve, promoting revascularization and enhancing nerve regeneration *in vivo* (75). **(D)** A PVDF/PLCL/PEDOT hydrogel with dual electroactivity promoted macrophage recruitment and M2 polarization, which stimulated Schwann cell activity to further drive nerve regeneration *in vivo* (86). This figure was created in BioRender. Shields, BR (2025) https://BioRender.com/nmrb11e .

The incorporation of metal-based ions into biomaterial scaffolds offers another approach for chemical immunomodulation. For example, magnesium ions integrated into a silk fibroin/alginate hydrogel matrix demonstrated significant immunomodulatory potential in a sciatic nerve transection injury model in rats (73). The presence of the magnesium-based nanoparticles led to release of magnesium ions, which enhanced the infiltration of M2 macrophages in the sciatic nerve injury (**Figure 2A**). The increased presence of pro-healing M2 macrophages enhanced SC recruitment to the injury site, which further improved remyelination and supported axonal outgrowth. Enhanced innervation increased the number and area of motor endplates, as well as promoted neuromuscular junction formation in the gastrocnemius muscle, which reduced muscle atrophy. This ultimately led to functional regeneration of nerve defects of up to 10 mm within 8 weeks (73).

### 5.2 Biological cues for peripheral nerve repair

Biological cues incorporated into biomaterials, including signaling molecules, cells, and cell-derived factors, serve as potent modulators of the immune response during peripheral nerve regeneration. Recent advances have demonstrated the efficacy of various material delivery systems for these applications. Immunosuppressive Tregs were encapsulated in a brain derived neurotrophic factor (BDNF)-chitosan hydrogel system, which preserved expression of Foxp3 in the Tregs and their proliferative potential. The preservation of the Tregs mitigated the harmful effects of inflammation, promoted revascularization, and enhanced nerve regeneration in a sciatic nerve injury in mice (74) (**Figure 2B**). Similarly, the delivery of fractalkine, a chemokine known for recruiting monocytes and activating microglia, has been explored in nerve injury models. The delivery of fractalkine through nerve conduits has demonstrated enhanced pro-healing M2 macrophage (CD206^+^CD68^+^) infiltration in the early stages of healing after injury, which dramatically increased the number of regenerating axons and enhanced nerve regeneration in large gap sciatic nerve injuries (76). Additionally, the delivery of cytokines, such as interleukin-4 (IL-4), have been explored for their regenerative potential. The delivery of IL-4 through polymeric nerve guidance channels supported the polarization of macrophages to their pro-regenerative phenotype, thus enhancing SC infiltration and migration, as well as improving axonal growth within the scaffold (77). Exosome-loaded hydrogel systems have also been developed to suppress macrophage infiltration and reduce proinflammatory factor expression. In one notable system, a soft hyaluronic acid methacrylate hydrogel provided rapid release of human umbilical cord-derived mesenchymal stem cell exosomes (uMSC-Exos). The rapid delivery of uMSC-Exos from the hydrogel inhibited proinflammatory macrophage infiltration and reduced the expression of IL-1α and TNF-α, promoting functional regeneration after sciatic nerve injury (78). In all these examples, a functional assessment of the gastrocnemius muscle was conducted to determine the relative effect of axon regeneration and muscle reinnervation. All examples demonstrated improved muscle functional outcomes that corresponded with the regenerating nerve.

### 5.3 Mechanical cues for peripheral nerve repair

Mechanical and physical cues, particularly scaffold topography and fiber alignment, play critical roles in modulating immune responses and enhancing cellular behavior after injury. Structured environments, such as aligned nanofibers, can direct immune cell behavior by promoting the polarization of macrophages toward the pro-regenerative M2 phenotype, while simultaneously enhancing Schwann cell recruitment, proliferation, and migration. These combined effects contribute to the development of a pro-healing microenvironment that supports axonal regrowth and tissue repair. Electrospun nerve conduits fabricated from poly[3(S)-methyl-morpholine-2,5-dione-co-lactic acid] [P(MMD-co-LA)] demonstrated the ability to provide sustained release of deferoxamine (DFO), an angiogenic, hydrophilic drug. These conduits not only accelerated human umbilical vein endothelial cell (HUVEC) migration and vessel tube formation, but also upregulated genes associated with angiogenesis, including VEGF; this promoted revascularization within the nerve defect and enhanced SC migration to support axon regeneration. The oriented nanofiber structure delivered physical signals that promoted macrophage polarization to their pro-regenerative M2 phenotype, resulting in increased infiltration of M2 macrophages *in vivo* and reduced expression of inflammatory factors within the injured nerve (75) (**Figure 2C**). Functional assessment of the gastrocnemius muscle resulted in significantly higher compound muscle action potential amplitudes, while histological analysis showed reduced muscle atrophy and larger muscle fiber diameter, indicating multi-tissue recovery as a function of nerve regeneration (75). Additional studies have shown that engineered conduits with controlled fiber orientation can enhance macrophage recruitment and M2 polarization, resulting in improved electrophysiological function, reduced muscle atrophy, and better sciatic functional index scores three months post-implantation (79). Aligned nanofiber scaffolds, including poly(L-lactic acid-co-ϵ-caprolactone)[ P(LLA-CL)] and aniline trimer-based polyurethane (A-PUAT), have similarly been shown to induce macrophage polarization toward a pro-regenerative M2 phenotype both *in vitro* and *in vivo*. This effect is supported by increased expression of anti-inflammatory markers such as CD206, Arg1, and IL-10, along with decreased expression of inflammatory markers Nos2, TNF-α, and CD80. The anti-inflammatory microenvironment enhanced Schwann cell proliferation, migration, and infiltration, ultimately supporting functional nerve and related muscle regeneration *in vivo* (80, 81).

### 5.4 Electrical cues for peripheral nerve repair

Electrically conductive biomaterials offer a unique approach to modulating immune responses after peripheral nerve injury. By replicating the natural bioelectrical properties of neural tissues, these materials promote macrophage polarization toward the pro-regenerative M2 phenotype, reduce inflammation, and enhance Schwann cell activity, creating a microenvironment conducive to axon regeneration and remyelination. For example, a biodegradable, waterborne polyurethane scaffold incorporating polydopamine-reduced graphene oxide (WPU-pGO) leveraged its conductivity to shift macrophage behavior toward an anti-inflammatory state, facilitating Schwann cell proliferation and axon extension. In addition to nerve regeneration, the WPU-pGO scaffold increased reinnervation, compound muscle action potential amplitude, and muscle mass in the gastrocnemius muscle (82). Similarly, electrically conductive polycaprolactone/silk fibroin composites have been demonstrated to promote M2 polarization and improve sacral nerve repair *in vivo* (83).

Advanced hydrogel systems have further demonstrated the benefit of conductive materials for nerve regeneration. Conductive hydrogels composed of polydopamine-modified silicon phosphorus nanosheets, gelatin methacrylate (GelMA), and decellularized ECM (SiP@PDA-GelMA/ECM) mimic the electrical environment of native nerve tissue, which facilitates macrophage polarization toward the pro-healing M2 phenotype. The biomimetic properties of the material, combined with sustained silicon ion release, promoted vascularization and enhanced Schwann cell differentiation in a peripheral nerve injury model, resulting in accelerated nerve regeneration and functional muscle repair in the gastrocnemius muscle (84). Other approaches, such as polyvinylidene fluoride (PVDF)-based nerve guidance conduits (**Figure 2D**) and PEDOT : PSS hydrogel bandages loaded with isopropyl alcohol (IPA), demonstrated similar immunomodulatory effects by enhancing M2 macrophage polarization, myelin formation, axon growth, and overall peripheral nerve repair, which supported functional muscle recovery (85, 86). Collectively, these conductive biomaterials highlight the potential of integrating bioelectrical and immunological signals to support functional peripheral nerve regeneration and resulting muscle repair.

## 6 Physiological overview of vascular regeneration

Although tremendous progress has been made in the development of immunomodulatory biomaterials to direct skeletal muscle repair and innervation, sufficient muscle and nerve function requires vascularization to sustain regenerating tissue. The formation of a vascular network is critical for interstitial fluid exchange of oxygen, nutrients, and waste in highly metabolic tissues like skeletal muscles, which must adapt to physiological demand. Blood vessels are composed of several different cell types. The internal lumen of blood vessels is lined with a layer of endothelial cells (ECs) known as the endothelium, which may be further supported by mural cells such as vascular smooth muscle cells and pericytes. As vessels increase in size from capillaries to arteries, these tissues take on more complex architectures to permit vasoconstriction and vasodilation amidst fluid transport, wherein the endothelium is surrounded by a thick layer of smooth muscle cells (referred to as the tunica media) and an outermost layer of connective tissue and elastic fibers (tunica adventitia) (87). In conditions such as ischemia, atherosclerotic clots impair sufficient perfusion to the extremities and disrupt this delicate architecture, and such progressive occlusion leaves skeletal muscle subject to muscle atrophy, pain, and weakness over time. Therefore, it is significant to highlight the key progenitor and immune cell populations which contribute to vascular formation, maintenance, and regeneration to best inform the design of immunomodulatory biomaterials to vascularize regenerating muscle.

### 6.1 Key progenitors

Angiogenesis is a highly coordinated and complex process which includes ECM remodeling, endothelial cell activation, lumen formation, and vessel stabilization by supporting mural cells, including pericytes and smooth muscle cells. During muscle regeneration, ECs activate and proliferate in tandem with MuSCs after injury and the vascular network expands its network of capillaries during the immune response (88). Several growth factors are associated with this cooperation between ECs and MuSCs, including VEGF, platelet-derived growth factor (PDGF), and basic fibroblast growth factor (bFGF), where the signaling of these factors may also be promoted by the presence of infiltrating immune cells. As such, timely modulation of immune cell behavior to coordinate endothelial cell activity is necessary to promote the growth of new blood vessels and enhance downstream tissue repair.

### 6.2 Notable immune cells

#### 6.2.1 Macrophages

Macrophages have been well-studied for their role in promoting angiogenesis and maturation of vessel networks across the spectrum of macrophage polarization states. In a study investigating the crosstalk of myogenesis and angiogenesis, it was found that macrophages activated by IL-4 and IL-10 stimulated the formation of lumenized capillaries via ECs and the differentiation of myotubes by muscle progenitor cells (88). Despite the previous belief that the angiogenic contributions of macrophages were reliant on a distinctive phenotype, Spiller et al. demonstrated that the coordination of M1 and M2 macrophages are required for vascularization of biomaterial scaffolds. Protein expression levels from each distinct phenotype coincided with different stages of vascularization: stimulants to prime endothelial sprouting were associated with M1 macrophages, maturation and recruitment of vessel-stabilizing pericytes with M2a, and remodeling protein secretion with M2c (89).

Several investigators have since sought to further investigate how vascularization is linked to macrophage dynamics. For example, Graney et al. delineated how human macrophage phenotypes (M1, M2a, M2c, and M2f) differentially and dynamically contribute to revascularization efforts by leveraging Transwell co- and tri-culture systems *in vitro*. M1 macrophages increased gene expression of markers for vessel sprouting and EC proliferation, potentially mediated by secretion of VEGF and TNF; with regards to M2 phenotypes, coculture analysis suggested that M2a macrophages coincided with increased EC-pericyte interactions, M2c macrophages contributed to regulating vessel sprouting, and M2f macrophages regulated vessel maturation. Furthermore, it was demonstrated *in vivo* that such M1/M2 macrophage phenotype diversity was critical for facilitating integration of engineered blood vessels with native host vasculature via subcutaneous implantation of HUVEC-embedded PLLA/PLGA porous sponge grafts in a mouse model. (90). In elucidating the coordination of macrophage dynamics in vascularization within the aforementioned studies, the shifts in protein expression levels and macrophage phenotype may be leveraged in muscle injury models—either through timed delivery of cytokines or biomaterial selection itself, e.g.—to best promote effective reperfusion in damaged skeletal muscle. In severe muscle injuries such as ischemia, understanding and capitalizing on how macrophages coordinate revascularization in the design of biomaterials for skeletal muscle repair can prove advantageous in salvaging damaged muscle.

#### 6.2.2 T cells

In addition to targeting cells of the innate immune system like macrophages, T cells have also been examined within the context of vascular regeneration. To analyze the roles of T cells in regenerating vascularized skeletal muscle, a systematic investigation of secreted factors from various CD4^+^ T cells (Th1, Th2, Th17, Treg) was conducted both *in vitro* and delivered from an injectable alginate biomaterial *in vivo* into a murine model of hindlimb ischemia. Among these subtypes, only conditioned media from Th2 and Th17 cultures significantly enhanced angiogenesis both *in vitro* and *in vivo* by directly enhancing endothelial sprouting, implicating potential cellular targets (91). Tregs have also been increasingly studied for their role in angiogenesis in ischemia models. In both murine and human models of peripheral arterial disease (PAD)—a condition associated with severe ischemic muscle injuries, Tregs potentiated neovascularization through upregulation of IL-10 and amphiregulin expression (92, 93). Specifically, diabetic patients with PAD demonstrated low levels of Tregs and high levels of CD4^+^ Th1 cells, contrary to normoglycemic controls, thus suggesting the dynamic and significant role Tregs may play in vascular repair. Thus, the paracrine signaling of IL-10 and amphiregulin from Tregs can serve as a therapeutic target to redress this imbalance and promote tube formation.

## 7 Design of immunomodulatory biomaterials for vascular regeneration

### 7.1 Chemical functionalization or base material selection

As with muscle and nerve repair, vascular regeneration relies heavily on immune cell interactions within the tissue microenvironment. Bioengineers have leveraged this understanding to design immunomodulatory biomaterials that chemically stimulate immune cell and vascular cell behavior, promoting neovascularization and the integration of vascularized grafts. In particular, scaffolds can be chemically modified with bioactive groups or peptides that engage immune cells and vascular progenitors to promote vascular regeneration. For example, a study compared three collagen scaffolds, specifically glutaraldehyde crosslinked, lipopolysaccharide (LPS)-soaked, and unmodified collagen scaffolds, implanted subcutaneously in mice to modulate the inflammatory response. The comparison of the three groups revealed that the moderately inflamed glutaraldehyde-crosslinked scaffold exhibited substantial blood vessel infiltration (89). Specifically, unmodified scaffolds induced a severe foreign body response matched with high levels of M2 macrophages and LPS-scaffolds demonstrated significant degradation by inflammatory macrophages; glutaraldehyde-crosslinked scaffolds exhibited a dynamic response by both M1 and M2 macrophages in hand with improved vascularization. Thus, this study emphasizes the need for a coordinated balance of macrophage phenotypes to support vascular morphogenesis.

**Figure 3:**
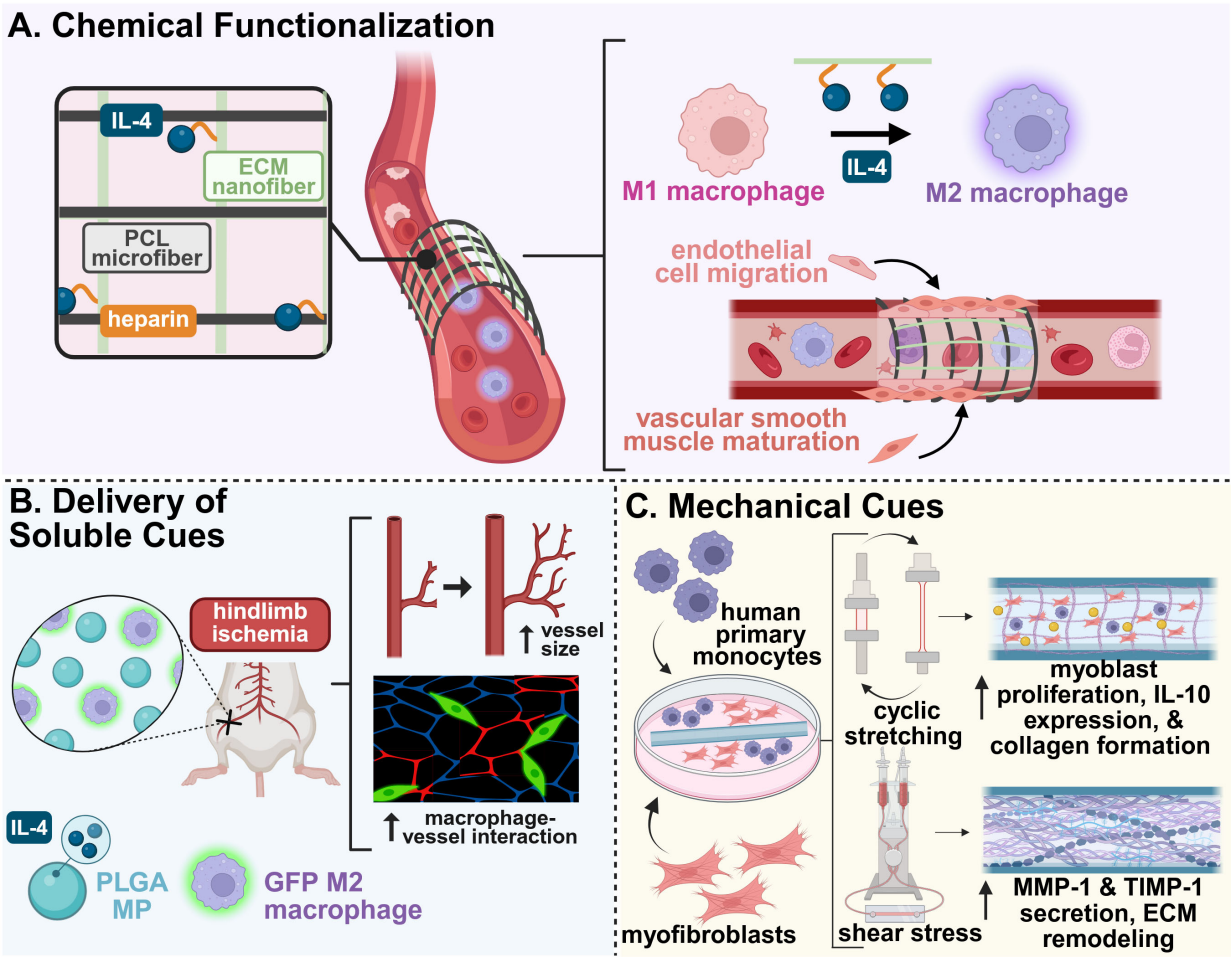
Example biomaterial strategies for modulating the immune system to promote vascular regeneration. **(A)** A hybrid electrospun scaffold composed of polycaprolactone (PCL) microfibers and heparin-functionalized placental ECM nanofibers carrying IL-4 enhanced M2 macrophage phenotype switching and downstream vessel formation and maturation when used as a vascular graft (95). **(B)** The codelivery of IL-4-loaded microparticles and exogenous, M2-stimulated macrophages promoted increased blood vessel size and macrophage-vessel engagement in a murine model of hindlimb ischemia (96). **(C)** An electrospun PCL-BU vascular scaffold co-cultured with human macrophages and myofibroblasts was exposed to cyclic stretch and shear stress for 20 days. Cyclic stretch promoted cell proliferation, increased expression of anti-inflammatory factors, and increased matrix deposition, while shear stress counteracted excessive tissue growth by enhanced matrix remodeling (97). This figure was created in BioRender. Mottel, L., Shields, BR (2025) https://BioRender.com/nmrb11e .

Several chemically functionalized scaffolds have demonstrated enhanced vascular outcomes through targeted immune modulation. For example, a synthetic, cell-free, electrospun scaffold functionalized with supramolecular stromal cell derived factor-1 (SDF-1α) peptide derivatives significantly increased CD68^+^ macrophage infiltration and vascularity in an *in situ* rat abdominal aortic model, while showing strong retention of human peripheral blood mononuclear cells *in vitro* (94). In a similar example, an electrospun hybrid scaffold with polycaprolactone (PCL) microfibers and human placental ECM nanofibers grafted with heparin and IL-4 successfully induced macrophage phenotype switching to their pro-regenerative M2 phenotype (**Figure 3A**). This induced a robust angiogenic response, including endothelial tube formation, vascular smooth muscle cell maturation, and ECM deposition, both *in vitro* and *in vivo* (95). Heparin-based chemistries have also been used to enhance bioactive molecule delivery. A poly(ethylene glycol) diacrylate (PEG-DA) hydrogel loaded with heparin provided sustained release of SDF-1α. The material increased monocyte recruitment in a dorsal skinfold window chamber model, enhancing vascularization (98). Collectively, these studies highlight how chemical modification of biomaterials can precisely orchestrate immune responses to support functional vascular regeneration.

### 7.2 Delivery of soluble immunomodulatory factors and cues

As discussed, vascular morphogenesis is a dynamic and tightly regulated process that depends on the spatiotemporal presentation of molecular cues to drive endothelial cell migration, proliferation, and stable vessel development. To enhance vascular repair, biomaterial strategies have focused on the localized and sustained delivery of immunomodulatory factors and soluble signaling molecules that target multiple stages of vascular development.

Several studies have demonstrated the therapeutic value of targeting specific immune cell populations with drug delivery systems. For example, the sustained release of lipid mediator aspirin-triggered resolvin (AT-RvD1) from PLGA films and PEG-MAL hydrogels promoted the accumulation of pro-angiogenic neutrophils at wound sites in full-thickness skin defect models, which induced a pro-regenerative macrophage response (99). Similarly, local delivery of FTY270 from PLGA films and nanofibers facilitated the recruitment of anti-inflammatory monocytes, leading to capillary and arteriole regeneration in murine spinotrapezius arteriole ligation model (100).

Building on insights from macrophage-driven angiogenesis, a biomaterial system was designed for sequential cytokine release. An initial burst release of IFN-γ, to promote M1 macrophages, was followed by a sustained release of IL-4, to support the transition to M2-macrophage phenotypes. This resulted in enhanced vascularization in bone scaffolds; however, this was largely mediated by early release of IFN-γ compared to late-stage presentation of IL-4 (101). Another strategy for improved vascularization was the co-delivery of IL-4-loaded microparticles (MPs) with exogenous M2-stimulated macrophages in a murine model of hindlimb ischemia (96). Although the administration of the IL-4 MPs alone significantly enhanced overall perfusion area, the delivery of both MPs and M2 macrophages increased average vessel size and promoted macrophage-vessel interactions (96) (**Figure 3B**). Furthermore, a dual-affinity PEG-DA hydrogel capable of co-delivering molecules of contrasting size and hydrophobicity, namely sphingosine analog FTY720 and SDF-1α, enhanced anti-inflammatory monocyte infiltration in the early stages of repair. The material prolonged the presence of CD206^+^ macrophages, which promoted microvascular remodeling, further promoting vascular regeneration in murine skin wound models (102).

Key players of adaptive immunity have also been targeted in the delivery of soluble cues. PLG scaffolds delivering ovalbumin (OVA) to ischemic muscle injury sites were used to leverage the adaptive immune response to support vascular regeneration. The delivery of OVA combined with vaccination of OVA with aluminum hydroxide (to induce Th2 OVA-specific T cells) led to the local recruitment of antigen-specific Th2 cells, significantly improving reperfusion, reducing tissue necrosis, and supporting myofiber regeneration in a hindlimb ischemia model. This approach demonstrated the potential of antigen depots to mobilize T cell responses in tissue repair (103).

### 7.3 Mechanical and physical cues

In addition to chemical and biological signals, mechanical and physical cues play a critical role in the design of biomaterials for vascular regeneration. Mature blood vessels are constantly exposed to dynamic forces such as shear stress and cyclic strain. Furthermore, the vessels’ structural and cellular compositions vary across vascular architectures. Therefore, biomaterials intended for vascular regeneration must be engineered to withstand physiological and mechanical loads while also providing biophysical cues that guide immune cell behavior towards tissue repair.

To reduce complications from scaffold failure associated with hemodynamic stress, a supramolecular elastomer scaffold paired with a bioreactor system was developed to investigate the effects of mechanical forces on immune cell behavior and tissue integration. Cyclic stretch was found to promote pro-inflammatory cytokine expression and matrix deposition, while shear stress counteracted these effects and enhanced MMP-mediated scaffold remodeling, demonstrating the dual role of these mechanical stimuli in modulating the immune response during repair (97) (**Figure 3C**).

Beyond application of external shear to promote vascular repair, other biophysical cues have been investigated. Early efforts by Madden et al. sought to design a proangiogenic scaffold as architectural templates for cardiac tissue integration. A poly(2-hydroxyethyl methacrylate-co-methacrylic acid) hydrogel was developed with parallel channels for cardiomyocyte organization; most notably this scaffold possessed micropores that demonstrated increased angiogenesis and reduced fibrotic response when implanted in nude rats, concomitant with M2 macrophage polarization (104). These scaffolds also demonstrated a pore size threshold necessary for neovascularization, parameters which may extend to the design of such biomaterials for skeletal muscle vascularization. The role of pore size on macrophage polarization was further analyzed to elucidate the foreign body response to biomaterial implants with different pore sizes in subcutaneous mouse tissue (105). Implants with 34 µm pores induced a slight foreign body response with a thin fibrous capsule but mostly cellular infiltrate, whereas those with an increased pore size of 160 µm possessed a greater fraction of fibrous tissue within its pores. Consequently, there was increased vascular density in smaller pore sizes compared to larger pores, with increased M2 macrophage signatures adjacent to these smaller pores (105).

## 8 Conclusions and outlook

There are a wide range of strategies for immunomodulatory biomaterials that possess significant implications for skeletal muscle regeneration, both in the context of direct muscle fiber repair and of promoting downstream innervation and/or vascularization (**Table 1**). Understanding the roles of tissue-specific progenitor cells and how they engage with innate and adaptive immune cells in response to injury is critical and necessary to inform the design of therapeutic biomaterials. Although not all of the biomaterials discussed were strictly utilized in a skeletal muscle injury model, the insight gained from these studies on advancing individual muscle, peripheral nerve, and vascular repair can be leveraged and translated into multi-tissue repair strategies. To achieve regeneration of complex tissue such as vascularized and innervated skeletal muscle, the integration and combination of several parameters discussed in this review (such as spatiotemporal presentation of soluble cues, electrical cues, and biomaterial identity) may promote optimal synergy between immunomodulatory and regenerative cellular targets going forward.

**Table 1:** Representative immunomodulatory biomaterials for vascularized and innervated skeletal muscle repair.

Tissue type	Biomaterial cue	Biomaterial type	Models	Targeted immune cells	Delivered factors	Source
Muscle	Biomaterial Identity	- Polyethylene (PE) particulate - Polyethylene glycol (PEG) particulate - Urinary bladder-derived extracellular matrix (ECM) particulate	Murine bilateral volumetric muscle loss injury	-Neutrophils -Macrophages	N/A	33
		Lymph node-derived ECM hydrogel	Murine volumetric muscle loss injury	Macrophages	N/A	35
	Antigen-Specific Responses	- Polyethylene (PE) particulate - Submucosal ECM	Murine bilateral volumetric muscle loss injury	Trauma-associated dendritic cells	N/A	41
		- Polycaprolactone (PCL) particulate - Submucosal ECM	Murine bilateral volumetric muscle loss injury	B cells	N/A	36
	Delivery of Soluble Cues	Matrix-bound nanovesicles (MBV)	Murine cardiotoxin muscle injury	Macrophages	IL-33+ MBVs	44
		Gold nanoparticles	Chronic muscle damage via microdamage or myotoxin injection	Macrophages	IL-4 or IL-10	39
Peripheral Nerve	Chemical Cues	Two-chambered chitosan film enhanced chitosan nerve guides (CFeCNGs)	Rat critical length sciatic nerve transection	M2 macrophages	N/A	71
		Silk fibroin/alginate hydrogel	Rat sciatic nerve transection	M2 macrophages	Magnesium ions	73
	Biological Cues	Polysulfone conduit filled with agarose hydrogel	15mm sciatic nerve gap injury in rats	CD206^+^CD68^+^ M2 Macrophages	IL-4 or Fractalkine	76
		Hyularonic acid methacrylate (HAMA) hydrogel	Rat sciatic nerve crush injury	M1 Macrophages	human umbilical cord-derived mesenchymal stem cell exosomes (uMSC-Exos)	78
	Mechanical Cues	Oriented nanofibrous poly[3(S)-methyl-morpholine-2,5-dione-co-lactic acid] [P(MMD-co-LA)] scaffold	Rat peripheral nerve defect model	M2 Macrophages	Deferoxamine (DFO)	75
		Electrospun aligned aniline trimer-based polyurethane (A-PUAT) conduit	Rat peripheral nerve transection model	M2 Macrophages	N/A	81
	Electrical Cues	Waterborne polyurethane/polydopamine-reduced graphene oxide (WPU-pGO) scaffold	Long gap sciatic nerve injury in rats	Macrophages	N/A	82
		poly(l-lactide-co-caprolactone) (PLCL), polyvinylidene difluoride (PVDF), poly(3,4-ethylenedioxythiophene) (PEDOT) (PVDF/PLCL/PEDOT) nerve conduit	Long gap sciatic nerve defect in rats	M2 Macrophages	N/A	86
Vascular	Chemical Functionalization	Hybrid electrospun scaffold (PCL microfibers and heparinized ECM nanofibers)	Rat abdominal artery defect	Macrophages	IL-4	95
		Heparin-containing PEG diacrylate (PEG-DA) hydrogel	Murine dorsal skinfold window chamber	Anti-inflammatory monocytes	Stromal derived factor-1α (SDF-1α)	98
	Delivery of Soluble Immunomodulatory Cues	- Poly(lactic-co-glycolic acid) (PLGA) film - PEG-Maleimide (PEG-MAL) hydrogel	Murine full-thickness skin defect	- Neutrophils - Macrophages	Aspirin-triggered resolvin (AT-RvD1)	99
		PLGA Microparticles	Murine hindlimb ischemia	Macrophages	IL-4 and exogenous, IL-4-stimulated macrophages	96
	Mechanical Cues	Electrospun PCL bis-urea (PCL-BU) scaffold	Bioreactor of (myo)fibroblast and primary monocyte coculture	Macrophages	N/A	97
		Poly(2-hydroxyethyl metahcrylate-co-methacrylic acid) hydrogel	Rat myocardial implant	Macrophages	N/A	104

Despite promising results in preclinical models, there are no clinical trials to our knowledge that have evaluated the efficacy of immunomodulatory biomaterials for innervated and vascularized skeletal muscle repair. For effective translation to the clinic, the long-term safety of a given immunomodulatory biomaterial, its degradation byproducts, and its long-term immunological host response must be screened during preclinical development and clinical validation. To meet regulatory requirements, there is also a need for potency assays for the manufacturing of these material systems; the development of these potency assays is challenging due to the complex mechanisms of action of these materials in modulating host immune responses. Although not discussed in this review, it is also significant for these emerging biomaterial strategies to address and account for varied demographics and cellular microenvironments across severe muscle injuries, particularly with regards to declined adaptive immunity with aging and the sex-based differences in immunity, which may uncover novel avenues and cellular mechanisms to explore. A potential strategy to address the complexity of varied injuries is to use omics technologies to probe inflammatory microenvironments, in order to inform AI models to provide deeper insights into individual immune profiles. Leveraging these tools can help delineate how immune responses differ across demographics, which can guide the design of personalized immunomodulatory regenerative therapies. Biomaterials engineered to fit a patients’ specific immune profile have the potential to enhance material integration, promote functional regeneration, and overcome current limitations of generalized therapeutic strategies, ultimately accelerating the clinical translation and availability of immunomodulatory biomaterial-based therapies.
